# Field Study on Indoor Thermal Environments of Monastic Houses and Thermal Comfort of Monks

**DOI:** 10.3390/ijerph20010008

**Published:** 2022-12-20

**Authors:** Chuan Chen, Mengshu He, Zihan Chu, Lishi He, Jiale Zhu, Yuan Bu, Jiangjun Wan, Lingqing Zhang

**Affiliations:** College of Architecture and Urban-Rural Planning, Sichuan Agricultural University, Dujiangyan 611830, China

**Keywords:** the severe cold region, monastic house, indoor thermal comfort, field research, adaptive predicted mean vote

## Abstract

Monastic houses are an essential part of the Tibetan monastic system in China. In this study, the monastic houses of Labrang in the Tibetan region of Gannan were used as the research objects. Physical parameters such as indoor temperature, humidity, and radiation temperature of the monastic houses were measured. The measured results were compared with the standard values, while the air temperature was linearly fitted using TSV, PMV, and aPMV. The results show that the temperature inside and outside the monastic houses fluctuates considerably; the theoretical thermal neutral temperature of the tested monks in winter is 22.46 °C, which is higher than the measured thermal neutral temperature in winter of 16.43 °C. When analyzing the results, it was found that the local climate, dress code, and the monks’ specific habits all impact the perception of thermal comfort, which creates a discrepancy between the accurate results and the standard values. The above findings provide a more comprehensive reference for the thermal comfort requirements of the monks in cold areas, which can be used as a guide for the improvement and evaluation of the monastic houses in cold areas.

## 1. Introduction

The need for comfort in the indoor thermal environment of a building is one of the basic human needs in a built environment. An excellent thermal comfort environment not only leads to satisfaction with the space, but also improves one’s physiological and psychological health [1]. China is a vast country with a rich climate and diverse cultures, and there have been many studies on the thermal comfort aspects of living and working spaces. Furthermore, most of the current research is based on different climate zones (severe cold, cold, hot summer and cold winter, hot summer and warm winter, and mild [2]), seasons, populations, and building functions. By climatic zone, studies include Tibet and Harbin in the cold zone [3,4], Xi’an, Tianjin, and Beijing in the cold regions [5,6,7], Chongqing, Nanjing, Chengdu, and Wuhan in the hot summer and cold winter regions, and Chongqing, Nanjing, Chengdu and Wuhan in the hot summer and cold winter regions [8,9,10]. Studies have also included other cities in the hot summer and warm winter regions [11] and Kunming in mild regions [12]. By season, studies have been conducted in summer [13,14,15], autumn [16], and winter [5,17]. By population, the relevant studies include early childhood [18], primary school students [13,19], university students [20], and elderly people [6,21]. In terms of building function, studies have been conducted on residential buildings [22,23], hospitals [24], kindergartens [25], nursing homes [26,27], office buildings [28,29,30], airport terminals [31] and high-speed rail stations [32]. Through a literature review, we found indoor thermal comfort studies related to almost every type of building with different functions. However, there are almost no studies on the thermal comfort of monastic Tibetan Buddhism communities in squatter buildings on the Tibetan plateau in China.

**Figure 1:**
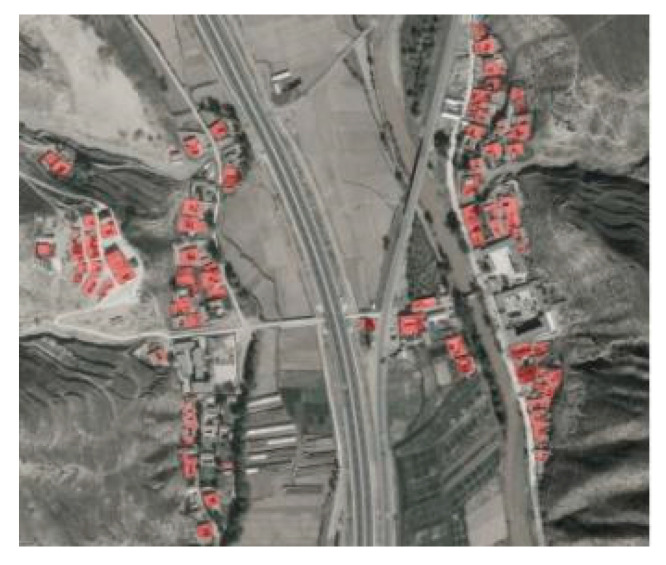
Map of the percentage of local rammed earth buildings (source: Google map).

Tibetan Buddhist temple complexes in China are broadly divided into the following three main functional areas: the Buddha area, the monastic area, and the Dharma area. Among them, monastic houses are places where monks live and are important examples of temple architecture, the forms of which mostly originate from local rammed earth dwellings (Figure 1). However, unlike traditional dwellings, monastic houses have their own specific functional space and limited means of thermal adaptation, due to regular working and resting schedules and the special biological/psychological needs of the monks. If not correctly designed, their indoor thermal environment can lead to poor health and reduced satisfaction with their use. In this study of monastic houses, we choose Labuleng Temple [33] in Gansu Province, China as the research object, and will investigate indoor thermal comfort in terms of both field surveys and model applicability, using a field survey methodology for residential buildings.

The most common indicators for assessing adaptive thermal comfort are predicted mean vote (PMV) and adaptive predicted mean vote (aPMV). PMV was introduced by Prof. Fanger in 1970. However, many existing studies have shown that the PMV index can differ significantly from the measured thermal sensory voting values in a naturally ventilated building environment, and is susceptible to external environmental influences. Maiti (2014) and Broday et al. [34] found in their study that PMV overestimates the actual subjective thermal sensation (TSV) response of Indian populations, which further expands the thermal comfort zone of these populations. Li et al. (2017) [35] indicated significant differences in the mean thermal sensation (AMTS) values obtained from a questionnaire compared to PMV calculated from field data in different cities. Occupants felt cooler on the cooler side of the city. Indraganti. (2013) et al. [36] showed that PMV always provides an overestimation of thermal sensations due to the wide range of adaptations of the subjects; Han, Yang et al. [37] showed that the average PMV calculated by the Fanger model was higher than the MTSV obtained from questionnaire data. A series of field studies using the PMV model for building thermal comfort have shown that ISO 7730, based on the thermal balance model, overestimated occupants’ thermal sensory responses on the ASHRAE scale at high temperatures and underestimated them at low temperatures [19,38,39,40,41,42,43].

After recognizing the differences in thermal expectations, behavioral differences, and physiological adaptations of living in different climatic zones for long periods, Professor Yao Runming [38] proposed the aPMV model. The aPMV model cohesively considered the effect of cultural, climatic, social, psychological, and behavioral adaptation on occupants’ thermal sensations. Instead of being the recipient of thermal stimuli, the occupant actively interacts with the environmental system through multiple feedback loops [44]. As the PMV model does not take into account real-world psychological and behavioral adaptations, aPMV accounts for the overestimation/underestimation of thermal sensation by PMV under warm and cool conditions separately [38]. In addition, the aPMV model helps us to investigate the dynamic thermal comfort temperature setpoint, one of the key factors that affects building energy consumption. Therefore, to improve the prediction accuracy of building thermal comfort in field studies, aPMV has been more widely used in thermal comfort studies in recent years [39,40,41,42,43,45].

Based on previous studies, combined with real-time feedback from the monks’ thermal sensations during the field survey, the main stages of this study were conducted as follows:

- Data on the temperature, humidity, and radiation temperature of the thermal environment in the monastic room were collected.
- The indoor thermal environment parameters of the building were measured by questionnaire collection of real-time thermal sensory feedback from the monks.
- The fitted curves of the TSV, aPMV, and PMV models were derived and compared for analysis to identify possible causes of the discrepancies.
- The measured data and survey results were analyzed to derive a neutral temperature and a thermal comfort temperature range for the monks, a group living in severe cold regions.

## 2. Materials and Methods

### 2.1. Study Area and Climate

**Figure 2:**
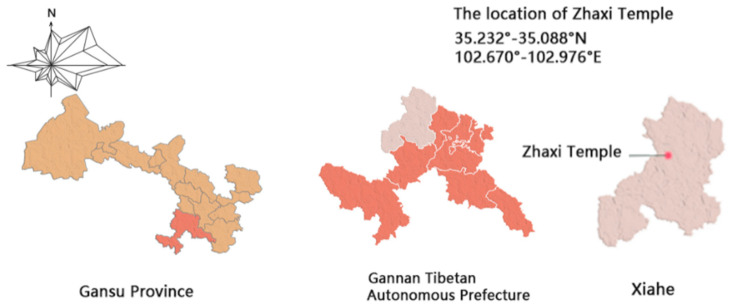
Location map (source: self-drawn).

**Figure 3:**
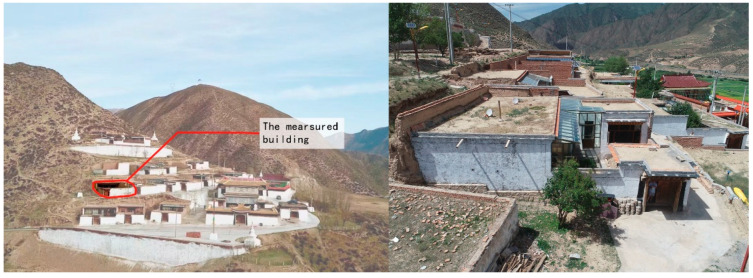
Current status of the Zhaxi Temple site (source: self-taken).

The Zaxi Monastery is located in the Tang Ga’ang Township, Xiahe County, Gannan Region, Gansu Province (Figure 2), with about 32 monks currently residing there, with an average age of twenty-two. The monastery is built on a slope, with the site high in the west and low in the east, and the dormitories are arranged in a stepped pattern according to the topography (Figure 3). The steep terrain of the Zhaxi Monastery has limited land and the dormitories are all one-story cottages, located in the middle of the monastery complex. The rammed earth building complex is a traditional rammed earth building, which is one of the important material expressions to create a unique style, display regional culture, and manifest ethnic characteristics in the Gannan Tibetan area. However, due to the cold and dry climate of Gannan and the age of the building, the outer wall of the squatter huts has different degrees of shedding and cracking, the surface of the building is uneven and the rammed earth structure is damaged. In order to adapt to modern life, passive sunroom technology has been introduced into construction of the traditional rammed earth squatter huts to improve the thermal insulation effect of the buildings, but there is no systematic design planning for the introduction of sunrooms, which leads to wasted floor space and poor indoor comfort.

**Figure 4:**
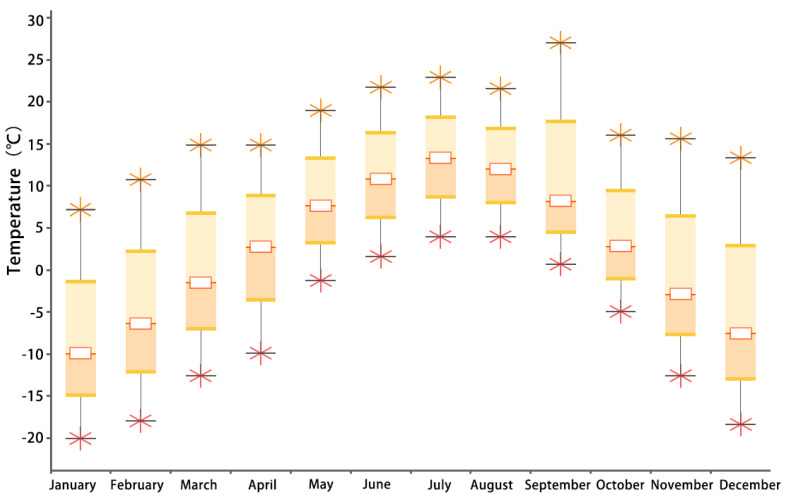
Box plot of monthly average temperature in the Xiahe County (source: Ecotect).

**Figure 5:**
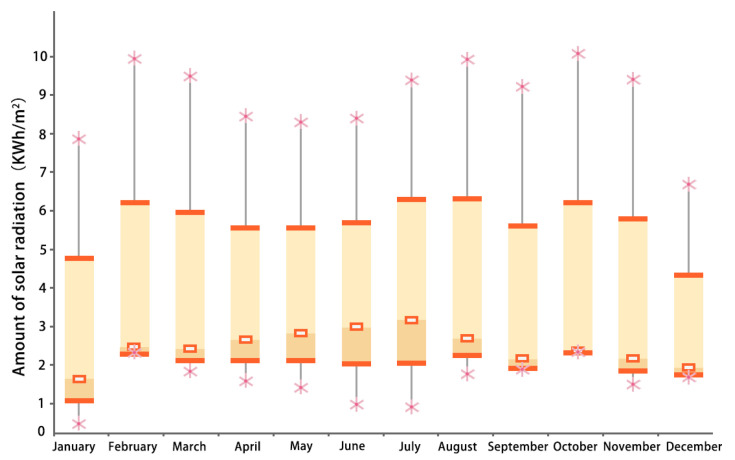
Monthly solar radiation box plot of the Xiahe County (source: Ecotect).

The Xiahe County of the Gannan Tibetan Autonomous Prefecture has a plateau-type climate with variable weather, a large temperature difference between day and night, and low temperature all year round. The coldest and hottest months are January and July, with average temperatures of −9.9 °C and 13.3 °C, respectively. The annual minimum temperature in the Xiahe County is about −10 °C, the annual maximum temperature is about 14.1 °C, and the annual average temperature is about 2.4 °C (Figure 4), which is the case in the severe cold region; at an altitude of 2910 m, the annual solar radiation can reach up to 3000 KWh/m^2^, the annual solar radiation can be as low as 1300 KWh/m^2^, and the annual average solar radiation is 2327 KWh/m^2^ (Figure 5), in an area with higher solar radiation.

### 2.2. Selected Architectural Features

**Figure 6:**
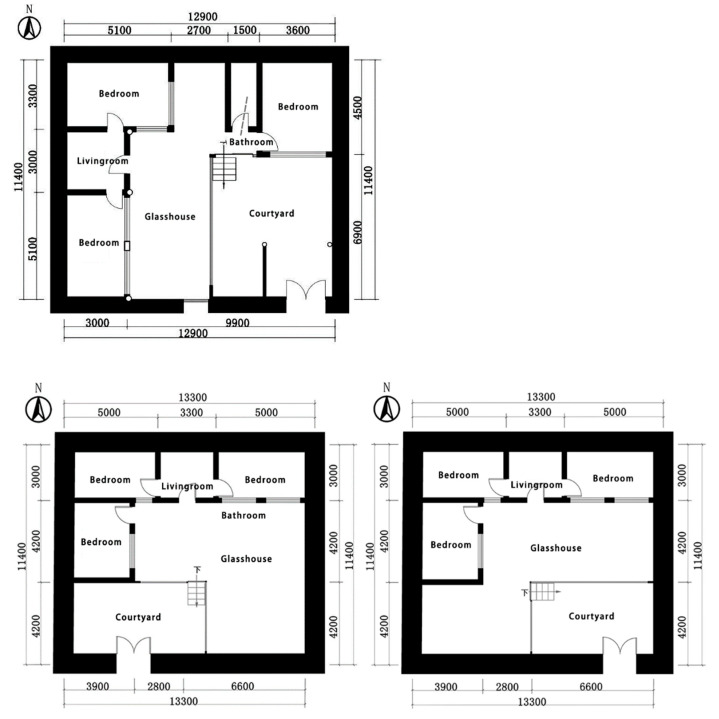
Plan of the monastery selected for measurement (source: self-drawn).

The monastery studied in this work includes a group of monastery buildings as part of the Zhaxi Monastery in the Tang Ga’ang township, which are all cottages with an area of about 147 m^2^ (Figure 6), and the number of monastery building residents is about 4. The building is a traditional rammed earth structure. The mountains face the west wall, and the rammed earth outer wall has good heat storage performance, but its strength and durability are poor. In addition, most of the monasteries are located on the west and north sides of the building, with the main monk’s room, living room, and ritualistic simmering furnace on both sides, and some of them have bathrooms. In this study, we mainly focus on monastery No. 1 attached to No. 18 (as shown in Figure 6 below).

### 2.3. Questionnaire Design

#### 2.3.1. Experimental Design

The site measurements were taken from 18 to 25 January 2021 and the field research was conducted based on ensuring the study life of the monks due to their special routine (Table 1). The investigators included 30 monks in the Zhaxi Monastery, with the oldest being 45 years old, the youngest being 6 years old, and the average age being 22 years old.

#### 2.3.2. Field Research

**Figure 7:**
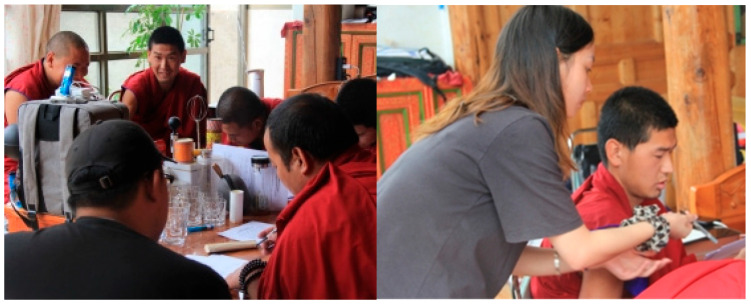
Picture of field measurements (source: self-taken).

Since the subjects were all Tibetans, they could not fully understand the meaning of the indices and scales. Therefore, the subjective questionnaire was produced in both Chinese characters and Tibetan languages, and the questionnaire options were prepared according to the Determination of PMV and PPD Indices for Medium Thermal Environments and Pro-visions for Thermal Comfort Conditions (GB/T 18049-2000), the Design Code for Heating Ventilation and Air Conditioning in Industrial Buildings (GB 50019-2015), and ISO7730 standards, and the research process was supplemented with interviews (Figure 7).

1. Subjective Investigation
  - The basic conditions of the subjects, including gender, height, weight, age, clothing amount, etc., were recorded and combined with the thermal environment parameters to calculate the PMV value.
  - The subjects’ thermal sensation in the monastic environment was obtained concerning ASHRAE 55-2004 and ISO7730 through the ASHRAE seven-level thermal sensation scale (Table 2); thermal acceptance and thermal comfort were recorded using the seven-level scale, and the specific scale division is shown in Table 2.
  - Subjects adapted to the squatter’s thermal environment through active heating methods, different heating times, etc., as well as their adaptive behaviors, such as increasing or decreasing the amount of clothing, activity level, etc.

**Table 2:** Thermal sensory voting division.

Time	+3	+2	+1	0	−1	−2	−3
**Hot Feeling** **Vote (TSV)**	Hot	Warm	Slightly warmer	Not cold, not hot	Cool	A little cold	It is too cold

2. Objective Investigation

In this study, a combination of horizontal and vertical surveys was used to formulate questionnaires for the subjects. A cross-sectional survey was conducted according to the daily routine of each subject, and a questionnaire survey related to thermal sensation and thermal comfort was conducted once every ten minutes and lasted for one hour during the monks’ free time. The time point of filling in the questionnaire survey should coincide with the time of the automatic recording of environmental parameters by the instrument, such as air temperature, air humidity, relative humidity, and black-bulb temperature. For the longitudinal survey, the winter questionnaire survey was conducted for three consecutive days for each subject, thus increasing the sample size and improving the accuracy of the findings at the same time.

### 2.4. Measured Parameters, Distribution Points, etc.

#### 2.4.1. Measured Physical Parameters

The measured thermal environment parameters inside the monastic room are as follows: average air temperature, average humidity, relative humidity, blackball temperature; the measured outdoor thermal environment parameters are air temperature, humidity and solar radiation intensity. The main testing instruments are the BTH01 temperature and humidity recorder, JTDL-4 four-channel thermal environment tester, JTNT-A multi-channel temperature, and heat flow tester and JTR04 blackball thermometer, which automatically records every 5 min.

#### 2.4.2. Location

**Figure 8:**
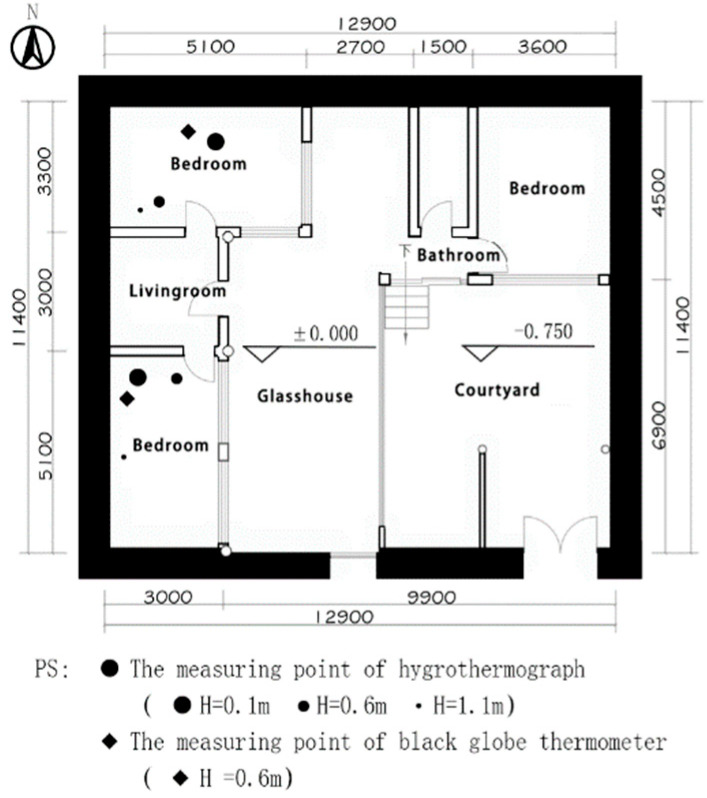
The layout of the measurement locations of the thermal environment parameters (source: self−drawn).

**Figure 9:**
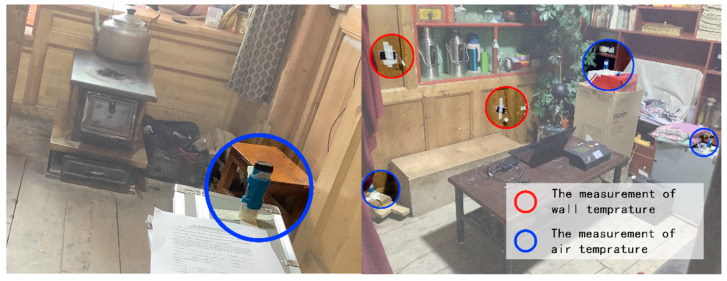
Picture of layout (source: self-drawn).

Since a stove heated the monastic room, the basic parameters of the environment were unevenly distributed. The subjects were in a sitting position when filling in the questionnaires; therefore, with regard to the JTJ/T347-2014 “Standard for Testing Methods of Thermal Environment of Buildings”, the air temperature and humidity testers were arranged at a horizontal distance of more than 0.5 m from the wall and a vertical distance of 0.1 m, 0.6 m and 1.1 m from the ground, respectively. The final measurement value was taken as the weighted average of the measurement values at different heights; the blackball thermometer was arranged at a vertical distance of 0.6 m from the ground; the solar radiation meter was arranged at a vertical distance of 1.1 m from the roof outside, with no shading. The arrangement of the measurement points of the thermal environment parameters is shown in Figure 8 and Figure 9.

### 2.5. Introduction of Indicators

The most common indicator is the predicted mean value of voting (PMV-PPD) proposed by Professor Fanger in 1970, based mainly on European human indicators [46]. The PMV indicator represents the thermal sensation of the vast majority of people in the same environment, and the PPD indicator represents the percentage of the population that is dissatisfied with the thermal environment.

The PMV-PPD indicator is based on the thermal comfort balance equation and takes into account the following six parameters: air temperature, relative air humidity, air velocity, MRT, human activity level and clothing conditions. Some data and regulations do not apply to specific practices, due to differences in climate characteristics, living habits, ethnographic characteristics, and adaptations in different places. For example, PMV is susceptible to external environmental influences in studies of PMV in naturally ventilated environments [47,48,49,50,51]. Humphreys and Nicol studied the effectiveness of PMV in everyday thermal environments and highlighted that PMV predicts higher thermal sensory voting in warmer environments compared to neutral environments [52]. Barger et al. showed that when PMV is applied to a naturally ventilated environment, it predicts higher thermosensory voting [44].

Therefore, Fanger and Toftum, using the “lower expectation” of the human body as the entry point for PMV model correction, used climate and buildings as two key factors that influence thermal expectations and corrected the calculated PMV by multiplying the calculated PMV value by the expectation factor e when the metabolic rate was low [53,54].

The literature has shown that the results of calculations using the recommended expectation factor “e” in some areas are satisfactory [55,56,57]. Hussein studied the thermal sensation in the hot and humid climate of southern Malaysia, where he found that 80% of the students found their indoor environment to be acceptable [57]. However, under the influence of the popularity of air conditioning, there are often cases where the actual cited values of the expectation factor are out of the expected range, and the PMV values derived from some regions using the recommended expectation factor also have large deviations from the actual human thermal sensation.

To fully explain the differences between the PMV model and the adaptive model and to establish a link between them, Yao Runming proposed the adaptive predictive mean vote (aPMV) based on the “black box” theory [38], which takes into account various factors that affect human thermal sensation, and used an adaptive factor “λ” with a different expectation factor [38]. The PMV model was modified, and the relationship between the PMV model and the adaptive theory was established by λ. Based on the aPMV model, Jiaping Liu found, in an adaptive thermal comfort study of primary and secondary school classrooms in winter in Northwest China, that the participants reported psychological adaptations to the cold environment because of the cold and harsh winter climate in their regions. In total, 90% of the participants had a comfortable temperature range of about 13.0–18.0 °C, which was significantly lower than the winter comfort temperature range recommended by the ASHRAE Standard 55-2017 (21.0–24.0 °C). This also suggests that previous models that did not account for physiological, psychological, and behavioral adaptations would predict higher thermal sensory polling [20].

China’s GB/T 50785-2012 Evaluation Standard for Indoor Thermal and Humid Environment in Civil Buildings, which is based on the aPMV model, establishes the evaluation standard for thermal comfort of naturally ventilated buildings in different building climate zones and accounts for the most critical index in the model, the adaptive coefficient λ.

This study aimed to improve the prediction accuracy of PMV using the adaptive predictive mean voting (aPMV) method proposed by Yao Runming et al. [38] to explore the relationship between the PMV model and adaptation theory, considering the physiological, psychological, and behavioral adaptation of monks to provide adaptive feedback. Finally, a thermal comfort model for monks in the Labrang Zaxi Monastery was derived to provide a research path and reference for monastic thermal comfort modification.

### 2.6. Equations Used in the Study

#### 2.6.1. Garment Thermal Resistance

According to the ASHRAE Standard 55-2004 for single-room clothing, thermal resistance values were estimated for the religious dress of monks in the survey area and the daily dress of general residents using public notices Equations (1)–(3) was calculated separately. During the survey, the additional seat thermal resistance was corrected by 0.15 clo, considering the influence of sitting posture on the garment thermal resistance.

(1) $$\mathrm{Men}:\;I_{clo}=0.113+0.727\sum I_{comp},$$

(2) $$\mathrm{Women}:\;I_{clo}=0.05+0.77\sum I_{comp},$$

(3) $$\mathrm{Monks}:\;R_{cl}=\left(t_{1}\right.-\left.t_{2}\right)/g$$

#### 2.6.2. Operating Temperature

In winter, the temperature of the inner wall surface of the envelope is low, and when the relative humidity is within the range of thermal comfort and the indoor wind speed is very low, the human thermal sensation is affected by both the indoor air temperature and the average radiation temperature, and the operating temperature $\;t_{op}$ should be used as the thermal comfort evaluation index. In the process of investigation, statistics of the field test data were obtained, and it was found that the average value of the indoor airflow rate was very low and was smaller than the minimum measurement value of the instrument, so Equation (4) was used to calculate the operating temperature, which means that the operating temperature $t_{op}$ is considered to be the average value of the air temperature ta and average radiation temperature $t_{T}$.

(4) $$t_{op}=0.5t_{a}+0.5t_{T}$$

#### 2.6.3. PMV Index and aPMV Index

Among the metrics used to evaluate indoor thermal comfort, the predicted mean vote (PMV) is more widely used. It uses the ASHRAE 7-level thermal sensory scale to predict the mean thermal sensation, and the analysis compares the subject’s TSV with PMV and APMV. The exponential equation used for PMV is Equation (5).

(5) $$PMV=\left(0.303^{e^{-0.036M}+0.0275}\right)\cdot \left(\left(M\right.-\left.W\right)-H-E_{c}-C_{res}-E_{res}\right)$$

In the study, the PMV index was calculated from the measured thermal environment parameters of individual clothing items and metabolic rate; APMV is the predicted value of thermal sensory voting obtained from people’s physiology, psychology, and behavior without artificial cold and heat sources.

## 3. Results

### 3.1. Environmental Parameters

**Figure 10:**
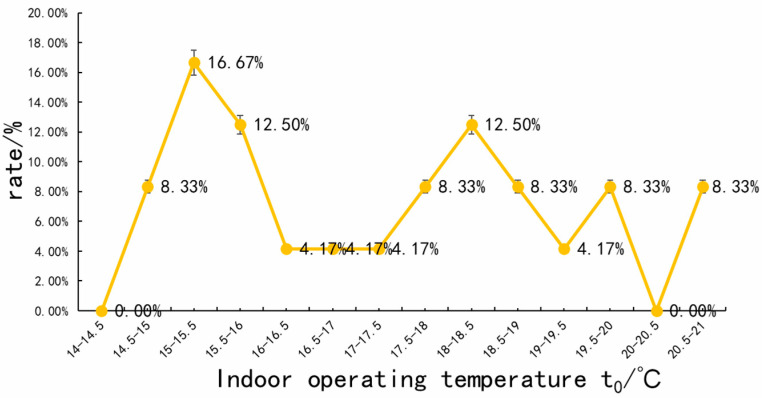
Frequency of the distribution of indoor operating temperature (source: self-drawn).

During the 39 valid sample periods measured, from 9:00 to 11:00, 14:00 to 16:00 and 19:00 to 23:00, the indoor temperature ranged from 15.8 to 20.3 °C, with an average temperature of 16.6 °C; the range of blackball temperature indoors ranged from 15.5 to 20.2 °C, with an average black-bulb temperature of 16.9 °C; the range of indoor air humidity was 23.7~44.1%, with an average humidity of 28.4%; the range of indoor operating temperature was 14.6~20.55 °C, with an average operating temperature of 17.3 °C; the range of the outdoor temperature was 2.3~6.8 °C, with an average temperature of 3.3 °C; the range of outdoor radiation temperature was 13.8~19.8 °C, with an average of 15.6 °C; the range of outdoor humidity was 17.1~36.6%, with an average humidity of 24.1%. The data analysis shows that the test area has a large temperature difference between the indoor and outdoor environments and has a dry climate. Due to the operation of the furnace heating equipment, the temperature difference between the indoor and outdoor environments was large during the test period, and the indoor temperature remained in the appropriate range, but the smoke from burning manure and coal meant that the air quality was poor. Table 3 provides data on various physical parameters that characterize the indoor climate, Figure 10 characterizes the frequency of the distribution of the indoor operating temperature $t_{op}$, and Table 3 provides data on various physical parameters that characterize the outdoor climate.

### 3.2. Garment Thermal Resistance and Metabolic Rate

**Figure 11:**
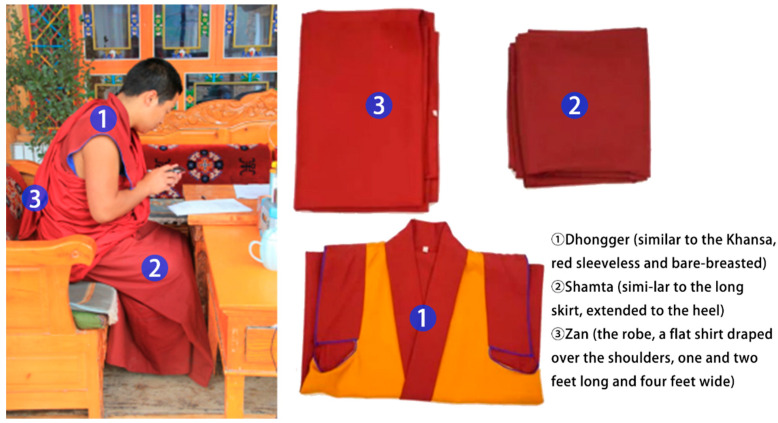
The dress (source: self-taken).

During the questionnaire survey, the subjects were sat in a sitting position, and very few of them were stood, while the clothing of the subjects was recorded, referring to the Indoor Thermal and Humid Environment Evaluation Standard for Civil Buildings (GB/T 50785-2012) [58]. The thermal resistance of the wooden seats was corrected according to the ASHRAE Standard 55-2004, and the thermal resistance of a single piece of clothing was expressed in clo (1 clo = 0.155 m^2^·K/W), using Equations (6) and (7) to calculate the total thermal resistance of garments for different age groups of interviewees. Their daily dress includes three pieces (see Figure 11), which are the Dhongger (similar to the Khansa, a red sleeveless garment that exposes the chest), the Shamta (similar to a long skirt, extended to the heel), and the Zan (a robe, with a flat shirt draped over the shoulders, one and two feet long and four feet wide). According to the clothing fabric and its material area and thickness, we consulted the literature to obtain the thermal conductivity of the clothing fabric, which was 0.08~0.13 W/(K·m). We used the thermal resistance Equations (8) and (9) to calculate the monks’ single piece of clothing thermal resistance, and then used the Equations (6), (8) and (9) to calculate the monks’ total clothing thermal resistance, which was 1.41 clo.

(6) $$\mathrm{Men}:\;I_{clo}=0.113+0.727\sum I_{comp},$$

(7) $$\mathrm{Women}:\;I_{clo}=0.05+0.77\sum I_{comp},$$

(8) $$\mathrm{Monks}:\;R_{cl}=\left(t_{1}\right.-\left.t_{2}\right)/g$$

(9) g=L/λ

$I_{clo}$ is the thermal resistance of a single person’s entire outfit, in clo.

$I_{comp}$ is the thermal resistance of a single garment, in clo.

$R_{cl}$ is the thermal resistance of a single piece of clothing for monks, in clo.

$t_{1},t_{2}$ are the temperatures of the monks on each side of the single garment, respectively, in °C.

g is the thermal conductivity of a single piece of clothing for monks, in W/m^2^.

L is the thickness of a single piece of clothing for monks, in m.

λ is the thermal conductivity of a single piece of clothing for monks in W/(m·°C).

**Figure 12:**
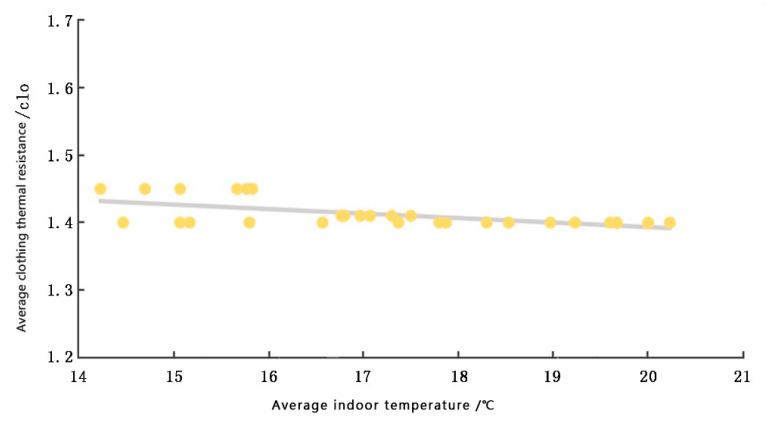
Indoor temperature–garment thermal resistance graph (source: self-drawn).

**Figure 13:**
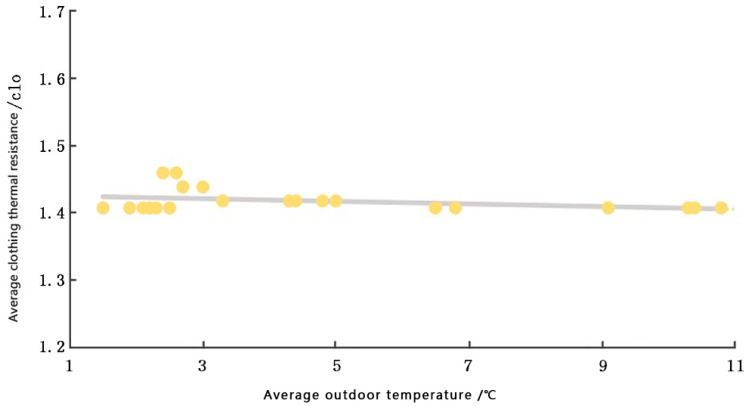
Outdoor temperature–garment thermal resistance graph (source: self-drawn).

The thermal resistance of the clothing of the interviewees was recorded and analyzed, and the relationship between the average thermal resistance of the clothing and the average indoor and outdoor temperature distribution was obtained, as shown in Figure 12 and Figure 13, respectively.

From Figure 12 and Figure 13, it can be observed that the average value of clothing thermal resistance of the interviewed participants is mainly concentrated in the 1.40 clo–1.5 clo range, and the average value of clothing thermal resistance is 1.457 clo. Influenced by their physical conditions and living habits, the average value of local winter clothing thermal resistance is lower than the average value of winter clothing thermal resistance in the general area. In the latter calculation, the metabolic rate was based on the activity status (leaning, sitting, standing, etc.) of the individuals at the time of the interview, concerning the GB/T50785-2012 Evaluation Standard for Indoor Thermal and Humid Environment in Civil Buildings [58]; the values were recorded as 0.8~1.4.

### 3.3. TSV, aPMV, and PMV

Every 7–10 people were used as a calculation interval in the order of ambient temperature from the lowest to highest. The average ASHARE 7-level thermal sensory voting values for subjects at different temperatures and heating conditions were obtained by controlling indoor humidity, wind speed and other variables and by varying the opening and closing of indoor doors and windows, and fireplace combustion, as a basis for studying the relationship between heat sensory values and ambient temperature. The temperature of each interval and the corresponding TSV, PMV, and APMV values were averaged, and the data of 120 interval samples were transformed to obtain 15 corresponding averages with 2 decimal places, as shown in the table below.

Since the indoor thermal environment tested fluctuated and was heated through a fireplace, PMV did not accurately predict the average thermal sensation of the monks. Yao Runming [38] proposed that the adaptive predictive evaluation of the thermal sensation (aPMV) model is more accurate and also explains the difference between TSV and PMV to some extent, so an attempt was made to predict the theoretical value of the results using aPMV. In the adaptive predictive mean thermal sensation model, aPMV is calculated as follows:

$$APMV=\frac{\mathrm{PMV}}{1+\lambda PMV}$$

where PMV is the predicted average thermal sensory index; λ is the adaptive coefficient (the value of λ reflects the level of adaptive regulation carried out by the human body or the degree of self-adaptation). The Zhaxi Temple is located in a cold region, according to the “Civil Building Indoor Heat and Humidity Environment Evaluation Standards” (GB/T50785-2012), resulting in a λ winter value of −0.5.

**Figure 14:**
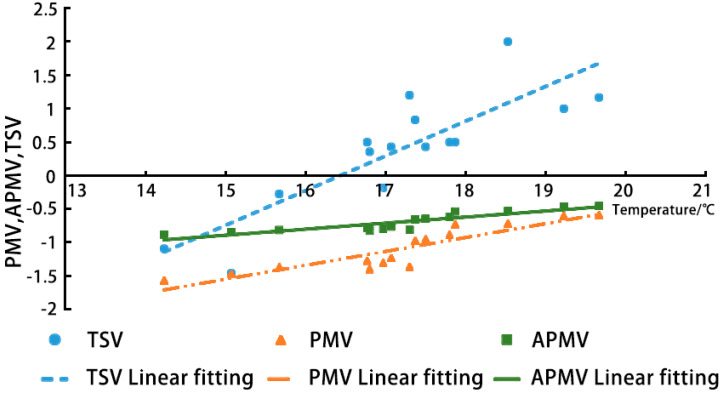
Linear fit of TSV, PMV, and aPMV values (source: self-drawn).

The TSV, PMV, and APMV values were fitted to the average indoor temperature, respectively, and the fitted curves shown below were obtained (Figure 14). The TSV curve is the curve of the measured thermal sensory voting value fitted to the indoor air temperature; the PMV curve is the curve of the predicted average thermal sensory index fitted to the indoor air temperature at the corresponding moment; the aPMV curve is the curve of the adaptive predicted average thermal sensory value fitted to the indoor air temperature.

The linear fits of TSV, PMV, and aPMV values to the operating temperature ($T_{a}$) were obtained after fitting 15 sets of sample polling values at different temperatures.

(10) $$TSV=0.5179T-8.5083\;\;\;R^{2}=0.7197,$$

(11) $$PMV=00901T-2.2395\;\;\;R^{2}=0.7959,$$

(12) $$APMV=0.2070T-4.6497\;\;\;R^{2}=0.8096$$

From Equation (10), it can be observed that the ambient temperature is 16.43 °C when TSV = 0, i.e., the corresponding measured thermal sensory neutral temperature is 16.43 °C.

From Equation (11), it is obtained that when PMV = 0, T = 24.85 °C, i.e., the theoretical thermal neutral temperature in winter is 24.85 °C, which is higher than the measured thermal sensory neutral temperature.

From Equation (12), it is obtained that when aPMV = 0, T = 22.46 °C, i.e., the adaptive mean thermal sensory neutral temperature is 22.46 °C, which is higher than the measured thermal sensory neutral temperature.

It is obvious from the fitted curve that the neutral temperature of aPMV is closer to the measured thermal sensation neutral temperature of TSV, verifying that there is a deficiency in the effectiveness of PMV, but this value still lies within the comfort range of the national standard, and there is evidence of a large deviation from the measured thermal sensation value.

Since the fitted curves of PMV, aPMV, and TSV all increased with increasing indoor air temperature, it indicates that the three directions were quite consistent. The TSV curve reached the thermoneutral temperature of 16.42 °C, indicating that the actual heat sensation of the subjects reached the most comfortable state at this temperature. The PMV values and aPMV values always reflected cold or colder human predicted heat sensations as the indoor temperature increased and satisfied PMV < aPMV, indicating that the predicted mean vote is smaller than the adaptive predicted heat sensation vote, i.e., the theoretical situation where a fireplace exists to improve the ambient heat exchange, but the effect is not significant.

However, as PMV is predicted from people’s psychological and physiological subjective heat sensations by the change in temperature and other heat source factors, aPMV is the predicted value of heat sensation voting obtained from people’s physiology, psychology, and behavior based on the absence of artificial heat and cold sources, and applies to residential areas where artificially created hot and cold environments are not relevant. The TSV results, when compared with the aPMV results, show that people will feel a little cold if they do not use the fireplace, and using the fireplace will result in neutral heat sensations.

In a further study of the actual heat sensation voting values of the subjects in the presence of a fireplace, the actual heat sensation comfort range tested was 15.67–17.87 °C, and 80% of the votes were “neutral”, with about 10% of the subjects considering the actual comfort level at this temperature to be “slightly hot”. The PMV predicted that the theoretical “neutral” feeling at this temperature was 0, and all the participants felt “slightly cold” or “cold” at this temperature. In the APMV prediction, the theoretical “neutral” vote at this temperature was 20%, and the remaining 80% of subjects felt “slightly cold”. However, since the TSV is the measured heat sensation, its representativeness is more in line with the actual sensation of the local monks, indicating that the prediction of the theoretical conclusion that the improvement of environmental heat exchange is not significant in the presence of a fireplace is influenced by the APMV. In contrast, the predicted value of the TSV curve for the temperature heat sensation compared to the PPD fitted curve is within more than 90% of the acceptable range for PDD voting, so the preferability of the acceptable prediction results is again exemplified, and also shows that the presence of a fireplace has a relatively significant effect on the change in the thermal environment.

In combination with the graphs, the theoretical prediction of PMV would require a temperature of 19.67 °C or more in the presence of a stove to achieve a ‘neutral’ perception of the subject. In comparison, the prediction of APMV needs to reach a temperature of 17.87 °C or higher to achieve the “neutral” perception of the subject. The actual heat sensation of the subjects that corresponds to this temperature is “slightly hot” and “hot”.

This shows that the predicted values of PMV and aPMV are not accurate in the case of indoor Tibetan fireplace heating, due to the influence of temperature distribution and thermal environment stability factors. Only the TSV curve can effectively demonstrate the trend of the thermal sensation of the monks who were in the monastic house. According to the TSV fitting curve, the overall fitting is good.

Analysis of the fitted curve of TSV shows that the value of −1 for T = 14.2 °C, which is the lower limit of the acceptable range, meaning that at the lowest temperature of 14.2 °C in a naturally ventilated building with a fireplace, the comfort of the monks in their residence is maintained. The thermal sensation felt by the monks when the weather was below this temperature at night was on the cold side, and the thermal sensation felt by the monks when the temperature reached its lowest (below 10 °C) at night was recorded as cold or extremely cold, which verifies the correctness of the results. When using the fireplace, the participants remained comfortable in the environment below the upper limit of 18.2 °C, while the TSV at this time was mainly concentrated between −1 and +1 (corresponding to an acceptable percentage higher than 90%); when the temperature was higher than the upper limit of 18.2 °C, the value corresponding to the TSV was greater than 1, and the use of the fireplace would result in thermal sensations on the hot side. The highest temperature in the actual test reached 19.7 °C, in the 18.2–19.7 °C range; the voting value was “slightly hot”, in line with the actual thermal sensation.

### 3.4. Heat Sensation and Wet Sensation

**Figure 15:**
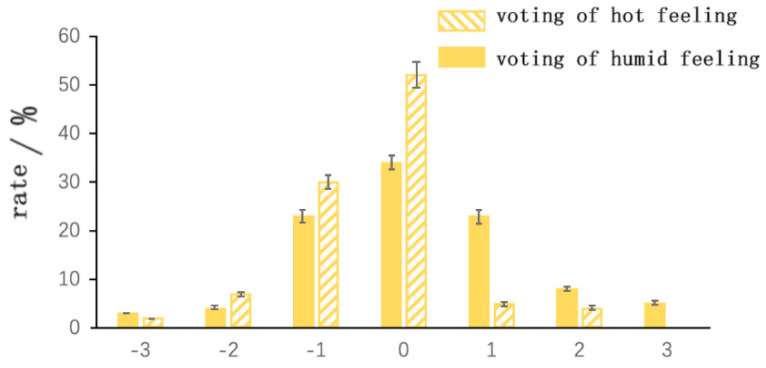
Frequency distribution of heat and humidity sensory voting (source: self–drawn).

The results of the wet and heat sensation surveys derived from the questionnaire are shown in Figure 15.

As shown in Figure 15, the respondents did not clearly show a linear or non-linear relationship for the wet sensation with changes in relative humidity. Possible reasons for this result are as follows: even though the researched area includes dry indoors and outdoors environments, i.e., the indoor relative humidity is generally below 30% and the outdoor relative humidity is generally around 30%, and can be as low as 10%, there was a high degree of personal physical acceptance of this by most of the residents, due to their longevity in the area, and nearly a third of the residents felt dry.

**Figure 16:**
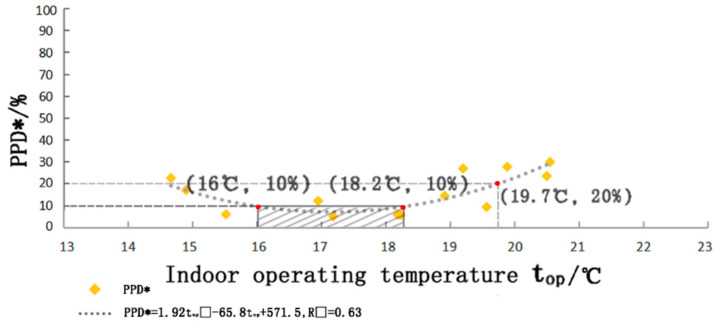
Indoor operating temperature graph (source: self-drawn).

As shown in Figure 16, the hot and cold sensations of those using fireplaces are relatively concentrated, with the majority of respondents feeling uncomfortably hot and favoring cooler or slightly warmer temperatures, indicating that the use of traditional fireplaces for heating is effective and has a high heating capacity in the local area. Likewise, the survey results show that nearly 20% of the respondents are uncomfortable.

It can be observed that in winter, when the relative humidity and temperature are low, due to the institutional conditions of the residents, 52% of the people who use the stove reported that the humidity feels just right and 33% of the people did not feel cold or hot, while the results of the survey show that 36% felt biased towards dryness. This result may be due to the following two reasons:

(1) The use of fireplaces can be effective for heating, but at the same time, it can increase the dryness of the local air humidity.

(2) The residents of the Gannan Tibetan area have been living in cold and dry climatic conditions for a long time and have adapted to these environments.

### 3.5. Thermal Acceptance Rate and Thermal Comfort Interval

We calculated the thermal unacceptability rate PPD* at a certain temperature (percentage of monks with thermal sensory vote values of −3, −2, 2, 3 of the total number of voters) and the PPD* rate for the room operating temperature, where $PDD^{*}=1.92t_{op}^{2}-65.8t_{op}+571.55$ and the correlation coefficient $R^{2}=0.63$.

As can be observed from Figure 16, the lower limit of acceptable temperature for 80% of the monks in winter is 13.8 °C and the upper limit is 19.7 °C, and the comfortable temperature range that 90% of the monks feel satisfied with is 16~18.2 °C. In addition, the comfortable temperature range accounts for about 31.6% of the indoor temperature distribution of the monastery building because the monastery will close the doors and windows tightly, the use of Tibetan-style stove heating will make the temperature of the monastic room rise gradually with the burning of the stove and exceed the comfortable temperature range, and it is difficult for the indoor thermal environment to meet the thermal comfort demand by artificial means; therefore, more suitable heating methods are needed for further improvement.

## 4. Discussion

(1) The mean values of garment thermal resistance of the interviewees were mainly concentrated in the range of 1.40 clo–1.5 clo, which is an improvement of 0.4–0.5 clo, compared to the winter garment insulation specified in the ASHRAE Standard 55-2017. Thus, the means of thermal adaptation adopted by the local monks in the test environment allowed them to be comfortable at lower room temperatures, and the local monks could accept a wider range of temperatures.

(2) PMV, aPMV, and TSV increase with the increase in indoor air temperature, while TSV is always higher than APMV and PMV within a certain temperature range, and the “scissor difference” phenomenon can be observed [22,59]. The combined PPD model fitting curves are indicative of the acceptability of the predicted results.

(3) During the survey period, both the local climate and indoor air were dry, and both the indoor and outdoor relative humidity were generally at or below 30%, but nearly two-thirds of the residents were accepting of this and wanted to keep the relative humidity low. Therefore, it is concluded that the local monks have adapted to the dry climate in their daily life and behavioral habits.

(4) In addition, the indoor thermal environment was somewhat improved by the alternating use of Tibetan fireplaces in the room during the test period, which also helped to improve their overall thermal perception [60,61], which led the tested monks to feel neutral in the colder environment and to be more accepting of the colder thermal environment. However, the continuous burning of the fireplace also leads to a continuous increase in indoor temperature and a continuous decrease in humidity, which can further affect indoor air quality and cause air pollution, and may even cause contribute to certain health risks [62]. Due to the limitation of the instrumentation, the measurement of CO_2_ concentration in the knot experiment is inadequate, and the correlation between the results and different indoor air quality needs further study. Based on the proposed adaptive and predictive evaluation of the thermal sensory model in this study, future work should be based on the “Double Carbon Scheme“ to explore clean energy or renewable energy heating measures.

## 5. Conclusions and Recommendations

### 5.1. Conclusions

(1) The average winter temperature of the Zhaxi Monastery is lower than −8 °C, and the average daily temperature difference between the indoor and outdoor environments fluctuates significantly. The monks that reside there have three specific dress codes—the Dhongger, Shamta, and Zan—with an average winter clothing thermal resistance value of about 1.41 clo, which is significantly higher than the thermal resistance value of winter clothing specified in the ASHRAE Standard 55-2017. The clothing chosen by the local monks is more adapted to the outdoor temperature.

(2) For the tested houses, the average operating temperature was 17.3 °C, and the operating temperature was influenced by the outdoor temperature. After continuous testing, it was found that the monks in the monastic houses would adjust the level and duration of the burning of the Tibetan fireplace to their own needs at different times, according to the changes in the room temperature. The theoretical thermal neutral temperature in winter was 22.46 °C at PMV 0, and the actual thermal neutral temperature in winter was 16.43 °C at TSV 0. The range of comfortable temperatures that 90% of the monks were satisfied with was 16 °C to 18.2 °C. The lower limit of the acceptable temperature for 80% of the monks was 13.8 °C, and the upper limit was 19.7 °C. The PMV was lower than the TSV, indicating that the PMV predicted colder actual thermal sensations and that there was some improvement in indoor temperature due to the fireplace, but the significance was low.

(3) The slope of the measured average heat sensation with temperature in the model is higher (0.52), that is, the monks are more sensitive to temperature. The reason for this result is that the monastery is in a severe cold climate zone with a large temperature difference between the indoor and outdoor environments. Although the monk community of the Zhaxi Monastery have lived here for a long time, they do not go in and out of the house frequently due to their special routines and have not fully psychologically adapted to the cold environment.

(4) In the environment of the Labrang Zaxi Monastery in the Tibetan region of Gannan, the actual winter heat neutral temperature for the local monks is 16.43 °C; however, 80% of the monks reported a wider range of acceptable temperatures, with a lower limit of 13.8 °C and an upper limit of 19.7 °C.

(5) The design values for the winter indoor thermal environment temperature of squatter buildings in harsh cold regions should take into account the local climatic conditions and fully consider the unique living and resting patterns, dress characteristics, psychological expectations, and physiological characteristics of the monastic community, and propose indoor thermal environment comfort standards that are suitable for the monastic community.

### 5.2. Suggestions

By analyzing the adequacy of solar energy resources in the Tibetan area of Gannan and focusing on enhancing comfort of monks at night in winter, the updated design strategy is proposed as follows.

(1) Building orientation: Most of the existing monasteries follow the original terrain and traditional layout concept of the main south orientation due to the mountains. According to the solar orientation of the Gannan area, the building orientation can be optimized to within 30° from southeast to the southwest to maximize the illumination time and solar radiation heat of the main function rooms.

(2) Architectural technology: One must make full use of the abundant solar energy resources in the Gannan Tibetan area, combine these resources with photovoltaic building integration, and form a self-circulating heating system through the addition of solar panels. Solar energy is stored during the day for conversion to thermal energy at night to ensure a comfortable indoor thermal environment in the building at night.

(3) Scale and material renewal: In contrast to traditional rammed earth forms, new materials could be mixed with an appropriate proportion of fly ash aggregates to chemically react with the rammed earth, thus further enhancing the robustness and stability of the rammed earth. Furthermore, the thickness of the rammed earth walls around the perimeter can be increased to 250 mm or more, thus effectively improving the thermal inertia and ensuring the even distribution of heat in the room.

(4) Layout integration: Based on the original shape, the restroom in the monastery could be placed near the end of the complex, which could reduce the heat dissipation brought by the airflow; therefore, the building is left with a certain gap on the side of the mountains to conduct heat, which can increase the illuminated surface for heat transmission. A sunroom could be set around the living room, and solar photovoltaic panels could be used to store and discharge energy, which can reduce the loss in heat transmission.

## Figures and Tables

**Table 1:** Monastic work schedule.

Time	Work Schedule	Time	Work Schedule	Time	Work Schedule
6:00	Get up	10:30–11:30	Concentration on scripture	14:00–16:30	Rest
6:30–9:00	Concentration on scripture	11:30–13:00	Have lunch	16:30–19:30	Concentration on scripture
9:00–10:30	Breakfast and rest	12:30–14:00	Lunch break	19:30–22:30	Dinner and rest

**Table 3:** Indoor and outdoor thermal environment parameters.

	Average Value	Standard Value	Maximum Value	Minimum Value
**Indoor** air temperature/°C	16.6	0.82	20.3	15.8
**Indoor** blackball temperature/°C	16.9	1.79	20.2	15.5
**Indoor** air humidity/%	28.4	7.11	44.1	23.7
**Outdoor** air temperature/°C	3.3	1.52	6.8	2.3
**Outdoor** blackball temperature/°C	15.6	2.14	19.8	13.8
**Outdoor** air humidity/%	24.1	7.28	33.6	17.1

## Data Availability

The raw/processed data required to reproduce these findings cannot be shared at this time as the data also forms part of an ongoing study.
